# Enhanced Functional Properties of Low-Density Polyethylene Nanocomposites Containing Hybrid Fillers of Multi-Walled Carbon Nanotubes and Nano Carbon Black

**DOI:** 10.3390/polym12061356

**Published:** 2020-06-16

**Authors:** Sandra Paszkiewicz, Anna Szymczyk, Agata Zubkiewicz, Jan Subocz, Rafal Stanik, Jedrzej Szczepaniak

**Affiliations:** 1 Department of Materials Technology, West Pomeranian University of Technology, Piastów Av. 19, 70310 Szczecin, Poland; sandra.paszkiewicz@zut.edu.pl; 2 Department of Technical Physics, West Pomeranian University of Technology, Piastów Av. 48, 70311 Szczecin, Poland; agata.zubkiewicz@zut.edu.pl; 3 Department of Electrotechnology and Diagnostics, West Pomeranian University of Technology, Sikorskiego str. 37, 70310 Szczecin, Poland; jan.subocz@zut.edu.pl; 4 Institute of Lightweight Engineering and Polymer Technology, Technische Universität Dresden, Holbeinstraße 3, 01307 Dresden, Germany; rafal.stanik@tu-dresden.de; 5 ELPAR Cable Factory, Laskowska str. 1, 21200 Parczew, Poland; jedrzej.szczepaniak@elpar.pl

**Keywords:** low-density polyethylene nanocomposites, hybrid carbon nanofillers, carbon nanotubes, nano carbon black, electrical conductivity, thermal conductivity

## Abstract

In this work, hybrid filler systems consisting of multi-walled carbon nanotubes (MWCNTs) and nano carbon black (nCB) were incorporated by melt mixing in low-density polyethylene (LDPE). To hybrid systems a mixture of MWCNTs and nCB a mass ratio of 1:1 and 3:1 were used. The purpose was to study if the synergistic effects can be achieved on tensile strength and electrical and thermal conductivity. The dispersion state of carbon nanofillers in the LDPE matrix has been evaluated with scanning electron microscopy. The melting and crystallization behavior of all nanocomposites was not significantly influenced by the nanofillers. It was found that the embedding of both types of carbon nanofillers into the LDPE matrix caused an increase in the value of Young’s modulus. The results of electrical and thermal conductivity were compared to LDPE nanocomposites containing only nCB or only MWCNTs presented in earlier work LDPE/MWCNTs. It was no synergistic effects of nCB in multi-walled CNTs and nCB hybrid nanocomposites regarding mechanical properties, electrical and thermal conductivity, and MWCNTs dispersion. Since LDPE/MWCNTs nanocomposites exhibit higher electrical conductivity than LDPE/MWCNTs + nCB or LDPE/nCB nanocomposites at the same nanofiller loading (wt.%), it confirms our earlier study that MWCNTs are a more efficient conductive nanofiller. The presence of MWCNTs and their concentration in hybrid nanocomposites was mainly responsible for the improvement of their thermal conductivity.

## 1. Introduction

It is well known that most of the polymeric materials exhibit electrically insulating properties. Notwithstanding, electrostatic dissipative or conductive behavior is required for many applications, i.e., antistatic housing applications, wire, and cable sheathing, or shielding against electromagnetic interference [1]. Electrically conductive polymers, like polypyrrole (PPy) or polyaniline (PANI), are found to be very expensive and relatively hard to process compared to that of other conventional polymers. Thus, a fairly common way in improving the electrical conductivity of the polymer composites is the introduction of conductive fillers or nanofiller into the polymer matrix. Nevertheless, the final properties of these (nano)composites are dependent on the content of (nano)filler. It is very important to improve the electrical and thermal conductivity of the polymer matrix while maintaining the balanced mechanical and processing properties. Wherein, a sharp transition from the insulating to the conducting behavior of composites occurs when the filler or nanofiller content reaches a critical value (so-called percolation threshold (Φ_C_) [2]), with only a slight increase in electrical conductivity at the further increase in filler content, the improvement of mechanical properties is a function of not only the share of nanoparticles, their proper distribution but also the effect on the morphology of the polymer matrix [3,4].

For several dozen years, carbon black (CB) has been applied as a convenient and inexpensive additive for thermoplastics and rubber materials in electrically conductive applications [5]. However, the amount that is required to observe the formation of conductive pathways through the insulating polymer matrices is much higher as compared to conductive fibrous fillers, like carbon nanotubes (CNTs) [5], etc. Nevertheless, the high content of these fillers or nanofillers (ca. 10 wt.% [6]) might cause the reduction of the other material properties like processability, gloss, and mechanical properties [1]. For this reason, CNTs are found to be very effective nanofillers in polymer matrices to obtain conductive materials at very low loadings [7], which is due to their superior electrical properties combined with the very high aspect ratio (AR), as high as 1000 for multi-walled carbon nanotubes (MWCNTs), which enables percolation at much lower concentrations if compared to CB [1]. Recently, CNTs were found to be excellent nanofillers for polymer-based (nano)composites not only to obtain antistatic or electrically conductive polymers but also to prepare (nano)composites with enhanced mechanical or other functional properties [4,8,9,10,11,12,13]. However, the price of CNTs, especially those which are not defected, with high specific area and AR, is still too high for many industrial applications. The other fillers, such as CB, carbon fibers (CFs) or carbon nanofibers (CNFs), and expanded graphite (EG), have generally much lower price, but their percolation threshold in polymers is usually much higher than that of the former ones. Therefore, the usage of the hybrid system of nanofillers in order to achieve the greatest improvement at the lowest concentration, thus the lowest price, are of economic interest. In such nanocomposites, the synergistic effects are desired, which means that the effect originating from the usage of the hybrid system of nanofillers is greater than the summarized effects of individual fillers [14,15] based on the percolation threshold of single fillers by adapting the excluded volume approach can be applied to discern the synergy. It was observed that the significant improvement of mechanical properties, electrical and thermal conductivity in polymer nanocomposites was dependent on the interfacial interactions of both nanofillers (interfacial adhesion) and also their compositions, i.e., the mass ratio of CNTs to CB and that higher ratios generally give rise to higher electrical conductivity in polymer nanocomposites [16,17,18,19,20,21,22].

In this work, the results of our continuous research are presented related to the development of new electrically conductive material based on hybrid carbon nanofillers, which can be used in cables as semiconductive screens. Resistivity of such nanocomposite shall not exceed 1000 Ω·m. Our earlier work was focused on low-density polyethylene (LDPE) nanocomposites containing MWCNTs and on the hybrid system being a mixture of MWCNT and graphene nanoplatelets (GNPs) as a conducive filler [16]. Therefore, in the present work one mixed nanofillers’ system consisting of MWCNTs and nCB, thus combining a nanofiller with a fiber-like shape (1D) with a spherical shape filler (3D). A potential synergistic effect of improving the functional properties of the hybrid LDPE nanocomposites resulting from a combined network between both carbon nanofillers was expected. Therefore, the physical, electrical, and thermal properties, the Stress-Strain behavior, and carbon nanofillers distribution in the LDPE matrix were investigated.

## 2. Materials and Methods

### 2.1. Materials

Low-density polyethylene (LDPE) (Malen E FGAN 23-D003, LyondellBasell, Germany) with a density of 0.922 g/cm^3^ and melt flow rate (MFR 190 °C/2.16 kg) of 0.31 g/10 min has been used as a polymer matrix in the obtained hybrid nanocomposites. The industrial thin multi-walled carbon nanotubes (MWCNTs, Nanocyl^®^ NC7000™) with a purity of 90% were delivered by Nanocyl SA (Sambreville, Belgium). The catalytic Chemical Vapor Deposition (CCVD) process was used for their synthesis. Multi-walled CNTs have an average diameter of 9.5 nm and length of 1.5 μm, density 1.75 g/cm^3^ [23], the surface area of 250–300 m^2^/g; electrical resistivity of 10^−6^ Ω∙m. Carbon black nanopowder (nCB) with the purity of >95% was purchased from US Research Nanomaterials, Inc. (Houston, TX, USA). nCB have average particle size of 150 nm, specific surface area >700 m^2^/g, content of ash: <3.2%, pH: 9.80, true density: 0.38 g/cm^3^ and resistivity of 30 × 10^−3^ Ω∙m.

### 2.2. Sample Preparation

The processing conditions for the materials used in this study were established by the evaluation of electrical resistivity measurements supported by scanning electron microscopy (SEM) analysis of LDPE/MWCNTs nanocomposites [16]. Besides, the parameters used in this study have been discussed in detail in the previous paper [16]. However, shortly: nanocomposites based on LDPE containing nCB, or the hybrid system of MWCNTs/nCB has been melt blended using twin-screw extruder (LSM30, Leistritz Laboextruder, Nuremberg, Germany) with closely occurring scrolls and interchangeable mixing sections, (diameter: D = 34 mm, L/D ratio = 23) equipped with two gravimetric feeders, a cooling bath, and a granulator. Three series of LDPE-based nanocomposites have been prepared: (i) nanocomposites containing nCB with concentrations of 3, 5, 7, 10, and 20 wt.%; (ii) nanocomposites containing hybrid system H1:1 being a mixture of MWCNTs and nCB in mass ratio 50:50, with the total concentration of 3, 5, 7, 10, and 20 wt.%; and (iii) nanocomposites containing hybrid system H3:1 being a mixture of MWCNTs and nCB in mass ratio 70:25 of MWCNTs and nCB, with the total concentration of 3, 5, 7, 10, and 20 wt.%. Similarly, as previously [16], in the beginning, the masterbatch (with the concentration of 20 wt.% of nanofiller/nanofillers) has been prepared which was then diluted to lower concentrations of nanofillers in the LDPE matrix. The following parameters were determined for extruding the masterbatch: feed zone: 20 °C, zone 1: 100 °C, zone 2: 170 °C, zone 3: 180 °C, zone 4: 190 °C, zone 5: 200 °C, zone 6: 210 °C, zone 7: 220 °C, and zone 8: 20 °C (nozzle). The rotational speed of screws of 40 rpm for masterbatches and nanocomposites containing 5–10 wt.% of carbon nanofillers and of 120 rpm for nanocomposites containing 1.5, 3 wt.% of MWCNT was used. Yield: 1.5 kg/h.

Subsequently, for mechanical, density, and thermal conductivity measurements the dumbbell shape samples (type A3, PN-ISO 37) were obtained by injection molding using Boy 15 (Dr BOY GmbH & Co., Neustadt-Fernthal, Germany) injection molding machine. The parameters of injection molding were determined following the guidelines of PN-EN ISO 294-1: 2017 standard, and the melting points of the materials determined from differential scanning calorimetry (DSC). The cut-out sections of the samples were used for thermal analyses.

The nanocomposite samples for SEM analysis were cryofractured in liquid nitrogen and subsequently coated in a vacuum with a thin gold film using thermal evaporation physical deposition method to provide electric conductivity.

Samples for electrical conductivity measurements were formed at the temperature of 190 °C and under pressure 5 bar (for 1 min) and 10 bar (for 1 min) by compression molding (Colin P200E, Dr COLLIN GmbH, Ebersberg, Germany) to the form of polymer foils with the thickness of ~250 μm. The thickness of thin foils was measured with a Micrometer Model No. 293–521 from Mitutoyo. Five measurements were taken for each sample, with an experimental error of ±0.001 mm. The thickness is an average value.

### 2.3. Characterization Methods

The density of the nanocomposites was measured by using the Archimedes principle on a high accuracy scale type AS made by Radwag (WPE 600C, Radom, Poland) at 23 °C, according to ISO 1183 standard. Before measurements, the hydrostatic balance was calibrated using standards of known density. Measurements were repeated five times for each sample. 

The melt flow index values (MFI) was used to study the influence of carbon nanofillers on the melt viscosity of LDPE. The measurements were performed on a CEAST Melt Flow Indexer (Pianezza TO, Italy) having a capillary with a diameter of 2.095 mm and a length of 8 mm. Tests were carried out according to ISO 1133 standard at 190 °C and under a load of 5.0 kg.

Analysis of the Stress-Strain behavior of the samples was performed using Autograph AG-X plus (Shimadzu, Duisburg, Germany) tensile testing machine equipped with a 1 kN Shimadzu load cell, an optical extensometer, and the TRAPEZIUM X computer software. Tests were carried out at room temperature with a grip distance of 20 m and using a constant crosshead speed of 5 mm/min. The values of the tensile modulus, tensile strength, and elongation at break of the nanocomposites were determined according to PN-EN ISO 527 standard. For each sample, five measurements were performed.

Scanning electron microscopy (SEM Hitachi SU-70, Tokyo, Japan) was used to analyze the dispersion of nCB and the hybrid system of MWCNTs/nCB in the LDPE matrix.

The electrical conductivity of the prepared nanocomposites was estimated based on the volume resistivity measurements which were performed according to the PN-EN ISO 3915 and PN-88/E-04405 standards [16]. Keithley Electrometer 6517A (Keithley Instruments, Inc., Cleveland, OH, USA) together with a set of Keithley 8009 was used for conducting the measurements of the volume resistivity of the 1 mm thick nanocomposite films.

The thermal properties of the nanocomposites have been analyzed as follows:

- differential scanning calorimetry (DSC) measurements were carried out using a DSC 204 F1 Phoenix (Netzsch, Germany) instrument. Measurements were proceeded under heating-cooling-the heating procedure in a temperature range from −25 °C to 200 °C under a nitrogen atmosphere at a flow-rate of 50 mL/min. Both heating and cooling rates were 10 °C/min. The temperatures and enthalpies of crystallization and melting were determined from first cooling and second heating scans, respectively. The heat fusion has been estimated by the integration of the area under normalized melting peak. The degree of crystallinity of the sample (X_c_) was calculated using the following equation:
  (1) $$X_{c}\;=\;\Delta H_{m}/\;\Delta H_{m}^{0}(1-\varphi_{n})$$
  where *ΔH_m_* is the melting enthalpy determined by DSC and $\Delta H_{m}^{o}$ (=293 J/g [24]) is the enthalpy of melting of fully crystalline PE, and φ_n_ is a weight content of nanofiller.
- thermo-oxidative stability of the pure LDPE and its nanocomposites was tested on the thermogravimetric analyzer (TGA 92-16.18 Setaram, Caluire-et-Cuire, France) in the temperature range from in the temperature range of 20–700 °C at the heating rate of 10 °C/min and under 20 L/min of synthetic air (N_2_:O_2_ = 80:20 vol. %).
- the thermal conductivity of the neat LDPE and nanocomposites was determined using the transient plane source (TPS) technique (Hot Disk TPS 2500, Uppsala, Sweden), and the Hot Disk thermal constants analyzer. The measurements were performed according to ISO 22007-2. Three tests were conducted, and the mean values were reported for each sample.

## 3. Results and Discussion

### 3.1. Physical Properties and Stress–Strain Behavior of the LDPE Nanocomposites

The influences of carbon nanofiller type and loading on density, melt flow index and tensile properties of the fabricated nanocomposites are shown in Table 1. The experimental density of nanocomposites was found to be slightly higher than the theoretical ones calculated by using the mixing rule. The addition of carbon nanofillers to the LDPE matrix affected the melt flow index (MFI). In our earlier study, we have observed a significant influence of MWCNTs on the rheological properties of LDPE/MWCNTs nanocomposites [16]. Their addition resulted in a significant increase in melt viscosity with the increase of CNTs loading. This increase of melt viscosity is a result of the existence of interconnected or network structures formed as a result of CNT-CNT and CNT polymer interactions [25]. As the content of MWCNTs increases, the LDPE-based hybrid nanocomposites showed lower values of the MFI because of the increase of their melt viscosity. In the case of LDPE/nCB nanocomposites with a lower nCB content (≤7 wt.%), a slight increase in the values of MFI was observed. The nanocomposites containing 20 wt.% of carbon nanofiller do not flow at 190 °C and MFI values were not able to evaluate.

**Figure 1:**
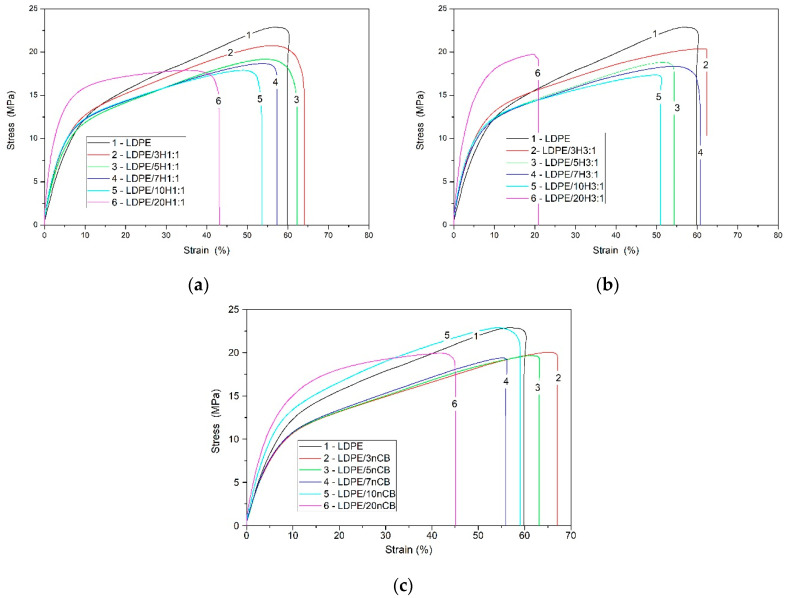
Representative stress-strain curves for hybrid nanocomposites LDPE/H1:1 (**a**), LDPE/H3:1 (**b**), and for LDPE/nCB (**c**) nanocomposites.

Figure 1 shows the representative stress-strain curves for neat LDPE and its nanocomposites. In Table 1 are presented the values of the Young’s modulus, tensile strength, and strain at break with their error bars (at 95 % confidence). It was found that the embedding of both types of carbon nanofillers into the LDPE matrix caused an increase in the value of Young’s modulus. Especially in the case of nanocomposites with the highest concentration of nanofillers (20 wt.%), a high stiffness can be observed. In the case of hybrid nanocomposites with 20 wt.% of carbon nanofillers, the Young’s modulus increased about threefold in comparison to the neat LDPE. Similarly, in the case of nanocomposites with nCB, an increase in Young’s modulus values was visible. However, comparing the results for nanocomposites with the same mass loading of nanoparticles, higher values of Young’s modulus were obtained for hybrid nanocomposites. Along with the increase of carbon nanoparticles’ concentration, the decrease in tensile strength for all nanocomposites was observed. Nanocomposites containing only nCB nanoparticles have slightly lower values of tensile strength and strain at break in comparison to the neat LDPE. The values of tensile strength at the same loading for hybrid LDPE nanocomposites are comparable in the error bars. At the same time, the values of strain at break for LDPE/H1:1 and LDPE/H3:1 nanocomposites with loading below 10 wt.% are within error bars, close to the value for neat LDPE, but at higher loading decreases. As shown in Table 1, a significant decrease of stress at break was observed for nanocomposites at the highest loading of carbon nanofillers. These results confirms our previous study of LDPE/MWCNTs [16]. The tensile strength of nanocomposites depends on many factors, among which the interphase adhesion between the reinforcements and the matrix, as well as the dispersion state, predominate.

**Figure 2:**
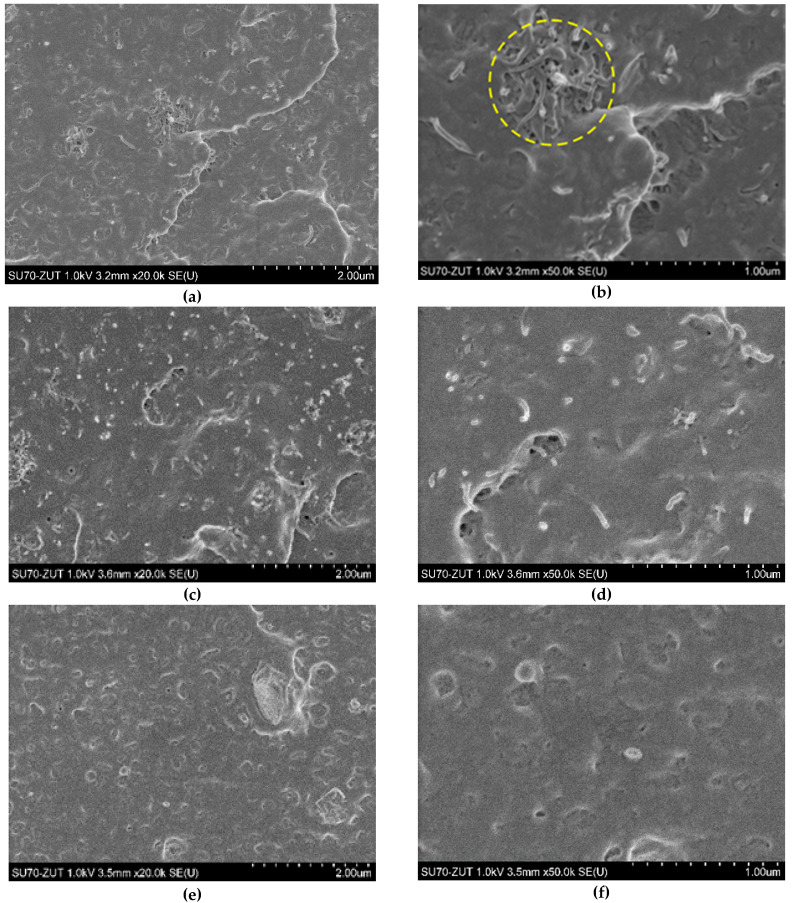
Scanning electron microscopy (SEM) images of (**a**,**b**) LDPE/5H1:1, (**c**,**d**) LDPE/5H3:1, and (**e**,**f**) LDPE/5nCB nanocomposites.

**Figure 3:**
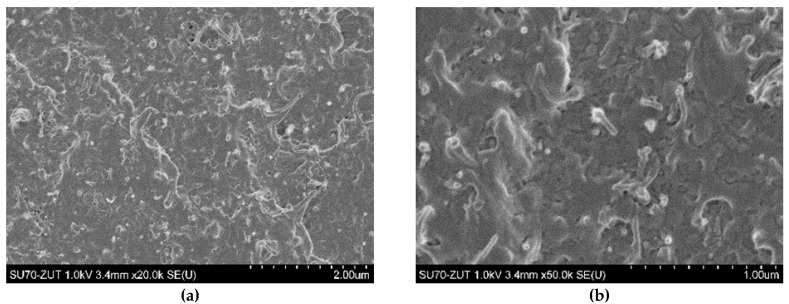

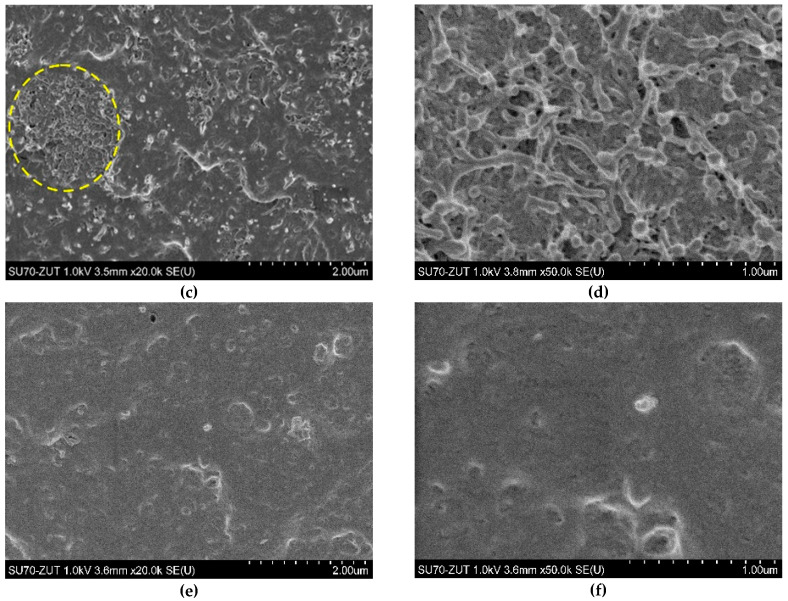
SEM images of LDPE/10H1:1 (**a**,**b**), LDPE/10H3:1 (**c**,**d**), and LDPE/10nCP (**e**,**f**) nanocomposites.

**Figure 4:**
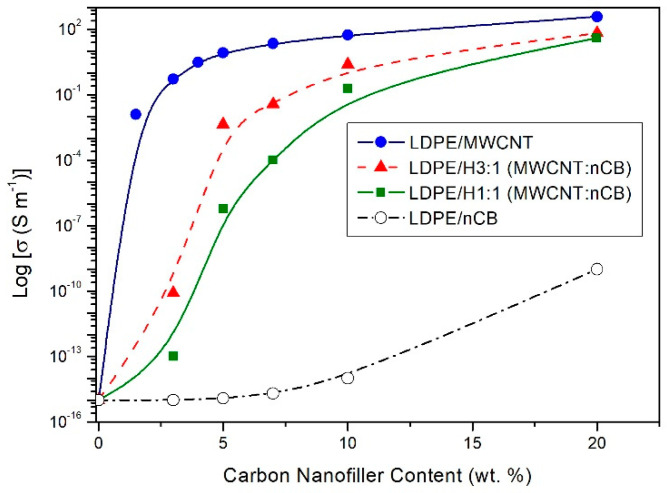
The electrical conductivity vs. nanofiller content (wt.%) of LDPE/multi-walled carbon nanotubes (MWCNTs) [23], LDPE/nCB, LDPE/H1:1 (MWCNTs:nCB), and LDPE/H3:1 (MWCNTs:nCB) nanocomposites.

The SEM analysis (Figure 2 and Figure 3) indicated that both of the used carbon nanofillers are rather homogeneously dispersed in the LDPE matrix but also at higher concentrations the agglomerates of MWCNT_S_ and nCB are incidentally present. Experimental and theoretical studies of the mechanical and electrical percolation in CNTs/polymer nanocomposites have shown that their tensile strength weakens at high filler loading, due to the aggregation of nanofillers and stress concentration, while the tensile modulus and electrical conductivity improve upon increasing of CNTs loading [26,27,28]. The investigated here LDPE nanocomposites shows such behaviour. The tensile strength and strain at break of the hybrid nanocomposites weakness with the increasing of hybrid nanofiller wt.% loading in LDPE, probably due to the increase of the CNT + nCB network density or to the aggregation of nanoparticles and stress concentration. Whereas the tensile modulus and electrical conductivity (see Figure 4) increase with the hybrid CNTs/nCB system or nCB loading. Besides, if in the nanocomposite, the high interfacial area and the strong interfacial interaction between nanofillers and polymer matrix occur, the third phase as interphase is formed [28,29]. This interphase significantly affects the mechanical properties of polymer nanocomposites. In investigated hybrid and nCB nanocomposites, we do not use functionalized carbon nanofillers, which could be responsible for enhancement of interaction between them and matrix molecules. A week interaction and poor wettability of used carbon nanostructures (MWCNTs, nCB) systems in LDPE may play an important role in reducing polymer-wrapping around multi-walled CNTs and nCB influencing on their tensile properties and electrical and thermal conductivity. The used thin and short MWCNTs have aspect ratio (L/D) of ~157, and nCB particles spherical in shape (average diameter 150 nm) have aspect ratio ≈ 1. Except week interaction/adhesion of CNTs (nCB) and LDPE matrix, the aspect ratio of the used carbon nanostructures can be responsible for observed difference in tensile strength of the resulting nanocomposites with increasing of their loading, especially at the loading of 10 wt.%. Usually, the high aspect ratio of CNTs provide the nanofillers network in polymer nanocomposites at very low loading [26].

### 3.2. Morphological Study

The degree of dispersion of the two MWCNT/nCB hybrid systems, which differ in a mass ratio (1:1 and 3:1), in the LDPE matrix was evaluated by SEM analysis. The representative SEM images of LDPE nanocomposites are presented in Figure 2 and Figure 3. In general, the MWCNTs and carbon black often tend to bundle together and form some agglomeration due to their high van der Waals attraction between the individual tubes/particles. It can be seen from Figure 2a,b, that at lower loading level (5 wt.%, above electrical percolation threshold—Figure 4) for both hybrid systems (H1:1 and H3:1) the MWCNTs and nCB nanoparticles are randomly dispersed into LDPE matrix. In hybrid nanocomposites, small agglomerates of entangled CNTs dispersed between individual nanotubes and CB nanoparticles are visible. At higher loading of the CNTs + nCB hybrid system, CNTs forms interconnected conductive network-like structures responsible for electrical conductivity (Figure 4) of these nanocomposites. At lower and higher loading levels, few bigger agglomerates of MWCNTs in the LDPE matrix were also observed (Figure 2b and Figure 3c). These agglomerates have a bigger size for hybrid nanocomposites containing MWCNTs and nCB in a mass ratio of 3:1. Figure 2e,f and Figure 3e,f show the effects of nCB particles content on the microstructure and morphology of the LDPE nanocomposites. Nearly spherical in shape nCB particles are uniformly embedded into the LDPE matrix. Most of them are separated without creating visible connecting chain-like networking structures, which are characteristic for conductive composites filled with CB. Besides, small inclusions with sizes bigger than 150 nm nCB are visible at higher magnification, this can indicate that some of them are agglomerated.

### 3.3. Electrical Properties

Figure 4 presents the electrical conductivity data for all prepared nanocomposites as a function of wt.% concentration of carbon nanofillers. The electrical conductivity of the nanocomposites containing the hybrid nanofillers’ system (MWCNTs + nCB) dispersed in the LDPE was compared with the electrical conductivity of LDPE nanocomposites containing only nCB or only MWCNTs [16]. Several key factors that can affect the electrical conductivity can be found, i.e., the appropriate degree of nanofillers’ distribution (dispersion), which due to the small size and high aspect ratio (AR), tend to form agglomerates or aggregates [30]. As previously described [16], MWCNTs cause a significant increase in electrical conductivity for about thirteen orders of magnitude with only 1.5 wt.% (percolation threshold Φ_c_) (Figure 4), which is a much lower value than those presented by other groups in PE [31,32]. Only, Du et al. [33] obtained for nanocomposites based on HDPE containing MWCNTs, the percolation threshold of about 0.15 vol. % (0.32 wt.%). However, they prepared HDPE/MWCNTs composites with a segregated network structure by alcohol-assisted dispersion and hot-pressing. Whereas, above the percolation threshold, only a slight increase in the value of electrical conductivity was observed along with a further increase of MWCNTs content. On the other hand, the incorporation of nCB did not significantly improve the electrical conductivity. Only the incorporation of 20 wt.% of nCB into LDPE enabled to observe the increase in the value of electrical conductivity for about six orders of magnitude. In general, to achieve percolation threshold, conventional conductive fillers such as CB, exfoliated graphite, and carbon fibers that are usually micro-meter scale materials, need to be added in content as high as 10–50 wt.%, resulting in a composite with poor mechanical properties and a high density. Therefore, herein, authors decided to apply carbon black nanopowder, which in intension would avoid the above-mentioned perturbances. However, nCB didn’t provide such a sharp increase in electrical conductivity at a lower concentration, probably due to the insufficiently large surface area or its surface properties. Such observations are in the agreement with the study of Lee et al. [34], who observed that when the CB content was less than 15 wt.%, the composites remained insulators, whereas increasing CB content resulted in decreasing resistivity. Besides, Wang et al. [35], obtained the percolation threshold at ca. 22 wt.% of CB in HDPE/CB nanocomposites prepared by melt mixing, although the final resistivity after the formation of a percolative path was about 10^2^ Ω·m. Referring to the percolation theory [36,37,38], the electrical conductivity of the final composite material strongly depends on the filler concentration. Moreover, the percolation threshold of composites/nanocomposites is greatly affected by the geometry of conductive fillers. Fillers with elongated geometry, (1D-type, such as fibers, tubes, etc.) can be used to achieve a relatively low value of percolation threshold due to their higher aspect ratio if compared to conductive particles with the sphere, ellipsoid, or other irregular shapes. For samples, containing a hybrid system of MWCNTs and nCB, the sharp increase in conductivity was observed at ca. 2.5 wt.% of the total nanofillers’ content. Since LDPE/MWCNTs nanocomposites exhibit higher electrical conductivity than LDPE/nCB or LDPE/MWCNTs + nCB nanocomposites with the same total nanofiller concentration, it implies that MWCNTs are a more efficient conductive nanofiller. This is in the agreement with our previous observations made for LDPE/MWCNTs + GNPs hybrid nanocomposites [16]. Similarly, as in the case of LDPE/MWCNTs + GNPs hybrid nanocomposites, the subsequent dilutions of the masterbatch (20 wt.%) to 10, 7, 5, and finally to 3 wt.%, (MWCNTs + nCB) show that the hybrids with the ratio of 3:1 MWCNTs:nCB demonstrated higher values of electrical conductivities in comparison to the one with the ratio of 1:1. Moreover, both series of hybrids exhibited values of electrical conductivity in between the values obtained for LDPE/MWCNTs and LDPE/nCB. For example, nanocomposites filled with 10 wt.% of the hybrid of MWCNTs and nCB with the mass ratio 3:1 and 1:1 have a mean electrical conductivity of 2.43 and 0.20 S/m, respectively. While the nanocomposites filled with 10 wt.% of MWCNTs and nCB have a mean electrical conductivity of 385 S/m and 10^−14^ S/m. Therefore, one can explain such behavior following the rule of mixtures. Unfortunately, one cannot observe the synergistic improvement in electrical conductivity in the case of the prepared hybrid. One can only make a conclusion, that according to the applicative character of CB in cable industry, the incorporation of MWCNTs, thus creating the hybrid of MWCNTs/nCB, might provide greater enhancement in electrical conductivity that CB itself, thus lowering the total content of nanofillers, final price of composites material without deterioration of processing and mechanical properties.

### 3.4. Thermal Properties

**Figure 5:**
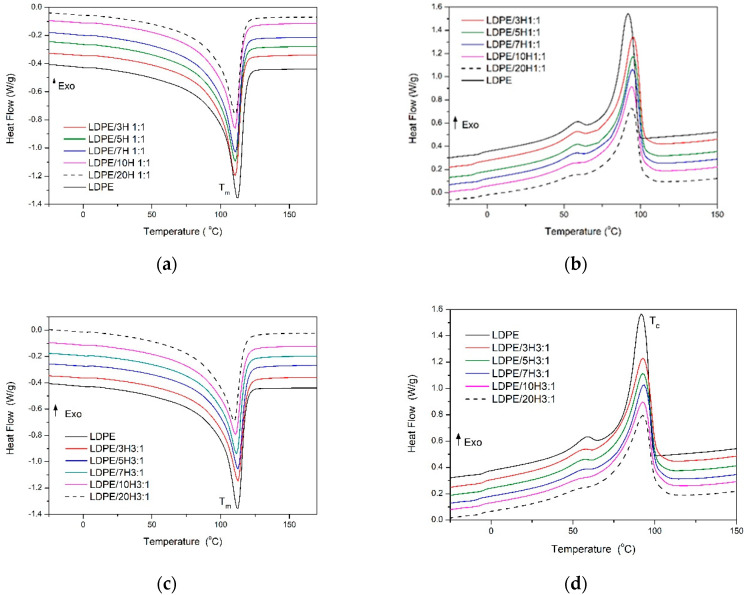

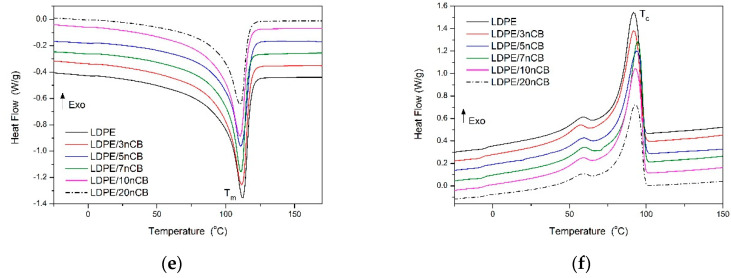
Differential scanning calorimetry (DSC) thermograms recorded during second heating and cooling for LDPE/H1:1 (**a**,**b**), LDPE/H3:1 (**c**,**d**), and LDPE/nCB (**e**,**f**) nanocomposites.

The thermal properties, by means of non-isothermal crystallization behavior, thermo-oxidative stability, and thermal conductivity of neat LDPE, “single” nanocomposites of LDPE/nCB, and hybrid nanocomposites of LDPE/MWCNT + nCB were studied. The DSC thermograms recorded during second heating and cooling in the temperature range of −25 to 200 °C are presented in Figure 5. Additionally, Table 2 summarizes phase transition temperatures and corresponding enthalpies of melting and crystallization, degree of crystallinity, and temperatures corresponding to 5 and 10 % of mass loss of LDPE-based nanocomposites during heating in an oxidizing atmosphere. From the second heating curves (Figure 5a,c,e) can be found that neat LDPE undergoes crystal melting at 112 °C, while the values of melting points (T_m_) within all three series of nanocomposites, i.e., hybrids at the mass ratios of 1:1 and 3:1 and nCB, did not change much (changes within 2 °C). Similarly, the cooling curves show that neat LDPE crystallized at 92 °C and the incorporation of nanofillers did not significantly affect the crystallization temperatures (T_c_) (Figure 5b,d,f). Moreover, the incorporation of carbon nanofillers caused almost no changes in the values of the degree of crystallinity (X_c_, Table 2). The observed changes in X_c_ were within the limit of measurement error.

The CNTs and CB usually paly a role of nucleating agents in accelerating crystallization in various CNTs (CB)/polymer systems [25,39,40]. However, CNTs exhibits different effects on chain mobility in different polymers. It was also reported that CNTs can generate also anti-nucleation effects, and super-nucleation effects on polymer matrices [41,42,43]. 

For the LDPE nanocomposites system investigated here, the used MWCNTs and/or nCB did not induce nucleation and increase crystallization rate of LDPE as nucleating agent did. Probably, due to the low value of surface energy and a poor wettability of CNTs and nCB, it is difficult for them to induce aggregation of polymer chains on their surfaces [44]. On the other hand, their presence in the molten LDPE can also hinder rearrangement of polymeric chains during crystallization.

**Figure 6:**
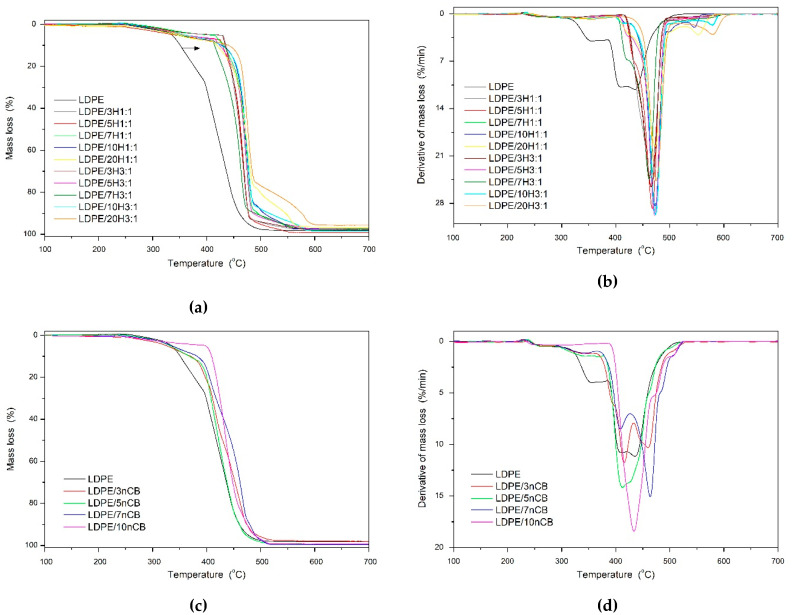
Mass loss and derivative of mass loss curves for LDPE/MWCNT + nCB hybrid nanocomposites (**a** and **b**, respectively) and LDPE/nCB nanocomposites (**c** and **d**, respectively) measured in an oxidizing atmosphere.

Polymer materials are generally used in an air environment. Therefore, from a practical application view, it is much more important to evaluate the thermal properties of materials in the air than in an inert atmosphere [38]. The thermal degradation behavior of LDPE-based nanocomposites in an oxidizing atmosphere is present in Figure 6, and the detailed data are summarized in Table 2. In general, the incorporation of carbon nanofillers, like CB or CNTs can improve thermal and thermo-oxidative stability of different polymer matrices [5,10,18,25,45,46,47,48]. As can be seen in Figure 6, the neat LDPE and nanocomposites in air atmosphere decompose in multistep process. It is well known that the oxidation of PE is a free radical-initiated autocatalytic chain reaction [48,49]. This reaction is slow at the beginning and accelerates with increasing concentration of the hydroperoxides. A process of oxidative degradation of LDPE and nanocomposites begins at temperature above 200 °C, when at first slightly increase of mass due to the oxygen absorption followed by the hydroperoxides formation was observed. Then at higher temperatures the clearly visible higher mass loss of the LDPE and nanocomposites attributed to decomposition of the accumulated hydroperoxides. Hydroperoxides decompose fast to reactive oxy and hydroxyl radicals, which are more reactive than peroxy radicals, and lead to the branching of the reaction chain, i.e., auto-acceleration of the degradation process [48]. β-scission of oxy macro-radicals yields carbonyl groups and other free alkyl radicals [48,49,50,51].

Mass loss and derivative of mass loss curves (Figure 6) of nanocomposites showed that the first steps of multiple decomposition of LDPE are strongly influenced by the presence of nCB and CNTs. Notably, thermal characteristic parameters (T_10%_ and T_50%_) shifted significantly to the higher temperatures by incorporation of nCB, and they increase along with the increase of nCB content. Similar behavior was previously observed by Wen et al. [52] in polypropylene (PP)/CB nanocomposites. For which it was found that the peroxy radicals could be efficiency trapped by CB at elevated temperatures to form a gelled-ball crosslinked network, which was responsible for the improvement thermal stability and flame retardancy of PP [52]. However, even more, the pronounced effect was observed when MWCNTs were incorporated into the system. Among all of the obtained series of nanocomposites, system 3:1 of MWCNTs: CB exhibited the highest values of T_10%_ and T_50%_. These results indicate that the incorporation of a hybrid system of carbon nanofillers is beneficiary from the point of view of the thermo-oxidative stability enhancement, which is in the agreement with our observations made for LDPE/MWCNT + GNP hybrid nanocomposites [16] and other polymer nanocomposites reinforced with the addition of CNTs [25]. The antioxidative activity of CNTs is a result of their radical scavenging ability [53]. The obtained here results confirm the antioxidant activity of the MWCNTs in preventing the oxidation of the polyethylene, which is beneficial for nanocomposites’ final properties.

**Figure 7:**
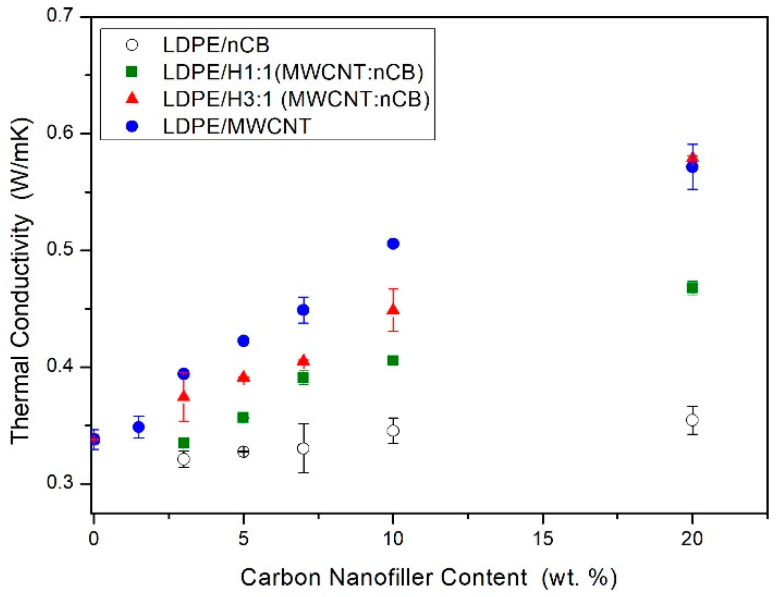
The thermal conductivity vs. nanofiller content (wt.%) of LDPE/MWCNTs [23], LDPE/nCB, LDPE/H1:1 (MWCNTs:nCB), and LDPE/H3:1 (MWCNTs:nCB) nanocomposites.

The observations made for the electrical conductivity enhancement are in good agreement with the thermal conductivity results. The thermal conductivity results of the prepared nanocomposites based on LDPE, measured by the hot disc method, are depicted in Figure 7. The thermal conductivity of all nanocomposites was found to increase with the addition of conductive particles thanks to the large thermal conductivity of the fillers [12,54,55]. Similarly, as in the case of hybrid nanocomposites based on LDPE containing MWCNT and GNPs [16], the incorporation of MWCNTs in the polymer matrix generated greater improvement in thermal conductivity in comparison to nCB. The lowest values of thermal conductivities were reported when nCB were mixed with the matrix, while the hybrid consisted of MWCNTs and nCB exhibited the values of thermal conductivity in between those obtained for “single” nanocomposite. Moreover, in the hybrid systems, it was observed, that the more MWCNTs were incorporated (hybrid 3:1), the higher values of thermal conductivity (Figure 7). One can, therefore, conclude, that this results from the rule of mixture, and unfortunately, no synergistic effect was observed. There are only a few studies that deal with the usage of a hybrid system of CNTs and CB to improve the thermal conductivity of the final materials [38,56], but none of those deals with LDPE-based nanocomposites. For instance, Song et al. [56] in the natural rubber nanocomposites filled with hybrid fillers of modified MWCNTs and CB, observed that the thermal conductivity of hybrid composites was improved by an average of 5.8% with 1.5 phr (phr—parts per hundred rubber) of modified MWCNTs and 40 phr of CB filled. A three-dimensional heat conduction network composed of hydroxyl CNTs and CB contributed to the good properties. The thermal conductivity of the hybrid composites increased as the temperature has risen. In turn, Hong et al. [45] observed that the thermal conductivities of the poly(dimethylsiloxane) (PDMS) based composites containing nano-sized carbon black (nCB) and carbon nanotubes (CNTs), linearly increased with filler concentration. The results present in this study are in the agreement with our previous paper [16] and with the observations made by Hong et al. [45] and suggest that CNTs are more effective in increasing the thermal conductivity of the polymer nanocomposites. In other words, the amount of nCB or GNPs [16] to enhance the thermal conductivity can be significantly reduced by the usage of a small number of CNTs. This result originates from the fact that the 1D-type nanofillers (MWCNTs) with a high aspect ratio and high electrical conductivity facilitates the percolation at lower concentrations. Since the thermal conductivity of the materials is achieved by both mobile electrons for conductors and phonons for insulators, the addition of highly conductive CNTs can greatly increase the thermal conductivity. This may be particularly important in the case of the cable industry, where it will allow to reduce the amount of carbon black to obtain electrical and thermal conductivity with balanced processing and mechanical properties.

## 4. Conclusions

Mixtures of MWCNTs and nCB in mass ratios of 1:1 or 3:1 were introduced into the LDPE matrix using a melt compounding method. In all mixtures, good dispersion and distribution of the carbon nanofillers were observed with SEM. The good dispersion of the carbon nanofillers mainly contributed to the higher Young modulus, which increased with the increases of carbon nanofillers. However, the addition of carbon nanofillers into LDPE caused a decrease in the tensile strength of the resultant nanocomposites with an increase in their concentration. The electrical and thermal conductivity of the obtained MWCNT + nCB hybrid nanocomposites were compared with results obtained for nanocomposites containing only nCB or only MWCNTs. No additional improvement of the nanocomposites mechanical and electrical and thermal properties was observed with addition of nCB in multiwalled CNTs and nCB hybrid nanocomposites when compared to LDPE/MWNCTs in terms of tensile strength, electrical and thermal conductivity, indicating no synergistic effect between nCB and MWCNTs. Nanocomposites with only MWCNTs showed higher electrical conductivity than nanocomposites containing only nCB or the mixed MWCNTs/nCB hybrid systems at the same total mass loading. This confirms that MWCNTs are a more efficient conductive nanofiller. Also, the thermal conductivity of hybrid composites was found to increase with the addition of MWCNTs in the hybrid ratio. TGA shows that the incorporation of a hybrid system of carbon nanofillers is beneficial from the point of view of the thermo-oxidative stability improvement of LDPE nanocomposites.

## Figures and Tables

**Table 1:** Physical properties of the neat low-density polyethylene (LDPE) and LDPE-based nanocomposites.

Sample	Carbon Nanofiller Content		d^t^	d	MFI (190 °C/5 kg)	E	σ_m_	ε_b_
	MWCT	nCB						
	wt.%	wt.%	g/cm^3^	g/cm^3^	g/10 min	MPa	MPa	%
LDPE	-	-	0.924	0.925	1.44	182.17 ± 10.12	22.93 ± 0.54	58.54 ± 2.74
LDPE/3H 1:1	1.5	1.5	0.937	0.931	1.41	256.90 ± 17.87	20.71 ± 0.16	64.23 ± 10.74
LDPE/5H 1:1	2.5	2.5	0.947	0.955	1.39	244.32 ± 20.98	19.39 ± 0.38	54.90 ± 9.93
LDPE/7H 1:1	3.5	3.5	0.956	0.961	1.06	279.75 ± 23.06	18.63 ± 0.17	56.59 ± 4.77
LDPE/10H 1:1	5	5	0.969	0.976	0.71	297.99 ± 21.17	17.60 ± 0.64	49.97 ± 4.04
LDPE/20H 1:1	10	10	1.014	0.998	NF	595.22 ± 47.43	17.89 ± 0.07	43.09 ± 1.66
LDPE/3H 3:1	2.25	0.75	0.938	0.932	0.781	298.42 ± 26.60	20.63 ± 0.14	61.33 ± 6.41
LDPE/5H 3:1	3.75	1.25	0.948	0.955	0.55	267.10 ± 16.21	18.94 ± 0.51	54.39 ± 5.60
LDPE/7H 3:1	5.25	1.75	0.959	0.965	0.29	266.23 ± 35.47	18.34 ± 0.15	60.63 ± 10.24
LDPE/10H 3:1	7.5	2.5	0.972	0.979	0.45	301.32 ± 28.71	16.88 ± 0.47	48.56 ± 7.48
LDPE/20H 3:1	15	5	1.021	1.007	NF	576.62 ± 58.23	19.75 ± 0.11	20.12 ± 4.19
LDPE/3nCB	-	3	0.907	0.942	1.48	191.48 ± 12.32	19.58 ± 0.50	64.74 ± 4.08
LDPE/5nCB	-	5	0.897	0.950	1.53	192.32 ± 19.68	19.33 ± 0.50	61.12 ± 9.10
LDPE/7nCB	-	7	0.886	0.956	1.50	197.31 ± 16.68	19.25 ± 0.56	55.87 ± 5.52
LDPE/10nCB	-	10	0.870	0.966	1.37	230.78 ± 13.98	21.85 ± 0.85	58.74 ± 8.41
LDPE/20nCB	-	20	0.825	0.876	NF	412.52 ± 14.21	19.92 ± 0.51	44.92 ± 3.81

d^t^, d—density calculated theoretically and measured at 23 °C respectively; MFI—melt flow index; NF—sample did not flow in these conditions; E—Young’s modulus; σ_m_—tensile strength; ε_b_—strain at break.

**Table 2:** The DSC and thermogravimetric analysis (TGA) data for the LDPE based nanocomposites.

Sample	T_m_ °C	ΔH_m_ J/g	T_c_ °C	ΔH_c_ J/g	X_c_ %	T_10%_ °C	T_50%_ °C
LDPE	112	131.1	92	130.5	45.4	351	418
LDPE/3H 1:1	112	128.5	94	127.3	45.8	436	461
LDPE/5H 1:1	111	121.4	95	120.4	44.2	429	463
LDPE/7H 1:1	111	120.4	95	120.5	44.8	432	469
LDPE/10H 1:1	110	112.5	94	112.9	43.2	428	471
LDPE/20H 1:1	110	100.2	94	101.0	43.4	419	472
LDPE/3H 3:1	111	126.4	95	127.1	45.1	434	462
LDPE/5H 3:1	112	118.8	93	117.8	44.2	424	469
LDPE/7H 3:1	111	114.8	93	114.0	44.1	415	456
LDPE/10H 3:1	110	99.6	93	98.9	43.0	426	472
LDPE/20H 3:1	112	129.8	94	128.9	44.9	441	476
LDPE/3nCB	111	120.7	94	120.5	41.7	370	430
LDPE/5nCB	111	122.4	94	122.0	42.3	368	422
LDPE/7nCB	111	121.7	93	120.5	42.1	382	445
LDPE/10nCB	110	101.5	90	99.8	43.9	409	436
LDPE/20nCB	112	131.1	92	130.5	45.4	-	-

T_m_—melting temperature; T_c_—crystallization temperature; ΔH_m_ and ΔH_c_—enthalpies of melting and crystallization, respectively; X_c_—degree of crystallinity, T_10%_—the temperature of 10 % mass loss, T_50%_—temperature of 50 % mass loss.

## References

[1] Pötschke P., Arnaldo M.H., Radusch H.J. (2012). Percolation behavior and mechanical properties of polycarbonate composites filled with carbon black/carbon nanotube systems. Polim. Polym..

[2] Stauffer D., Aharony A. (1994). Introduction in Percolation Theory.

[3] Farrukh M.A. (2016). Functionalized Nanomaterials.

[4] Krishnamoorti R., Vaia R.A. (2007). Polymer Nanocomposites. J. Polym. Sci. Part B Polym. Phys..

[5] Bhattacharya M. (2016). Polymer nanocomposites—A comparison between carbon nanotubes, graphene, and clay as nanofillers. Materials.

[6] Huang J.C. (2002). Carbon black filled conducting polymers and polymer blends. Adv. Polym. Technol..

[7] Bauhofer W., Kovacs J.Z. (2009). A review and analysis of electrical percolation in carbon nanotube polymer composites. Compos. Sci. Technol..

[8] Chen H., Ginzburg V.V., Yang J., Yang Y., Liu W., Huang Y., Du L., Chen B. (2016). Thermal conductivity of polymer-based composites: Fundamentals and applications. Prog. Polym. Sci..

[9] Paszkiewicz S., Szymczyk A., Janowska I., Jedrzejewski R., Linares A., Ezquerra T.A., Wagner H.D., Tenne R., Rosłaniec Z. (2017). Comparative study on the properties of poly(trimethylene terephthalate)-based nanocomposites containing multi-walled carbon (MWCNT) and tungsten disulfide (INT-WS2) nanotubes. Polym. Adv. Technol..

[10] Rahmat M., Hubert P. (2011). Carbon nanotube-polymer interactions in nanocomposites: A review. Compos. Sci. Technol..

[11] Sahoo N.G., Rana S., Cho J.W., Li L., Chan S.H. (2010). Polymer nanocomposites based on functionalized carbon nanotubes. Prog. Polym. Sci..

[12] Spitalsky Z., Tasis D., Papagelis K., Galiotis C. (2010). Carbon nanotube–polymer composites: Chemistry, processing, mechanical and electrical properties. Prog. Polym. Sci..

[13] Ma P.-C., Siddiqui N.A., Marom G., Kim J.-K. (2010). Dispersion and functionalization of carbon nanotubes for polymer-based nanocomposites: A review. Compos. Part A Appl. Sci. Manuf..

[14] Paszkiewicz S. (2014). Polymer hybrid nanocomposites containing carbon nanoparticles. Situ Synthesis and Physical Properties.

[15] Sun Y., Bao H.D., Guo Z.X., Yu J. (2009). Modeling of the electrical percolation of mixed carbon fillers in polymer-based composites. Macromolecules.

[16] Paszkiewicz S., Szymczyk A., Pawlikowska D., Subocz J., Zenker M., Masztak R. (2018). Electrically and thermally conductive low-density polyethylene-based nanocomposites reinforced by MWCNT or hybrid MWCNT/graphene nanoplatelets with improved thermo-oxidative stability. Nanomaterials.

[17] Zhou S., Hrymak A., Kamal M. (2018). Effect of hybrid carbon fillers on the electrical and morphological properties of polystyrene nanocomposites in microinjection molding. Nanomaterials.

[18] Li L., Zhang M., Ruan W. (2015). Studies on synergistic effect of CNT and CB nanoparticles on PVDF. Polym. Compos..

[19] Paszkiewicz S., Szymczyk A., Sui X.M., Wagner H.D., Linares A., Ezquerra T.A., Rosłaniec Z. (2015). Synergetic effect of single-walled carbon nanotubes (SWCNT) and graphene nanoplatelets (GNP) in electrically conductive PTT-block-PTMO hybrid nanocomposites prepared by in situ polymerization. Compos. Sci. Technol..

[20] Paszkiewicz S., Szymczyk A., Pilawka R., Przybyszewski B., Czulak A., RosŁaniec Z. (2017). Improved thermal conductivity of poly(trimethylene terephthalate-block-poly(tetramethylene oxide) based nanocomposites containing hybrid single-walled carbon nanotubes/graphene nanoplatelets fillers. Adv. Polym. Technol..

[21] Chen J., Du X.-C., Zhang W.-B., Yang J.-H., Zhang N., Huang T., Wang Y. (2013). Synergistic effect of carbon nanotubes and carbon black on electrical conductivity of PA6/ABS blend. Compos. Sci. Technol..

[22] Baltá Calleja F.J., Bayer R.K., Ezquerra T.A. (1988). Electrical conductivity of polyethylene-carbon-fibre composites mixed with carbon black. J. Mater. Sci..

[23] Kim S.H., Mulholland G.W., Zachariah M.R. (2009). Density measurement of size selected multiwalled carbon nanotubes by mobility-mass characterization. Carbon.

[24] Wunderlich B., Doyle M. (1957). Specific heat of synthetic high polymers. VIII. Low pressure polyethylene. J. Polym. Sci..

[25] Szymczyk A. (2012). Poly(trimethylene terephthalate-block-tetramethylene oxide) elastomer/single-walled carbon nanocompsites: Syntheis, structure, and properies. J. Appl. Polym. Sci..

[26] Li H., Zare Y., Rhee K.Y. (2018). The percolation threshold for tensile strength of polymer/CNT nanocomposites assuming filler network and interphase regions. Mater. Chem. Phys..

[27] Chen Y., Pan F., Guo Z., Liu B., Zhang J. (2015). Stiffness threshold of randomly distributed carbon nanotube networks. J. Mech. Phys. Solids.

[28] Zare Y., Rhee K.Y. (2020). Analysis of the connecting effectiveness of the interphase zone on the tensile properties of carbon nanotubes (CNT) reinforced nanocomposite. Polymers.

[29] Rostami M., Mohseni M., Ranjbar Z. (2012). An attempt to quantitatively predict the interfacial adhesion of differently surface treated nanosilicas in a polyurethane coating matrix using tensile strength and DMTA analysis. Int. J. Adhes. Adhes..

[30] Thostenson E.T., Li C., Chou T.W. (2005). Nanocomposites in context. Compos. Sci. Technol..

[31] Zhang Q., Rastogi S., Chen D., Lippits D., Lemstra P.J. (2006). Low percolation threshold in single-walled carbon nanotube/high density polyethylene composites prepared by melt processing technique. Carbon.

[32] Ciselli P., Zhang R., Wang Z., Reynolds C.T., Baxendale M., Peijs T. (2009). Oriented UHMW-PE/CNT composite tapes by a solution casting-drawing process using mixed-solvents. Eur. Polym. J..

[33] Du J., Zhao L., Zeng Y., Zhang L., Li F., Liu P., Liu C. (2011). Comparison of electrical properties between multi-walled carbon nanotube and graphene nanosheet/high density polyethylene composites with a segregated network structure. Carbon.

[34] Lee J.-H., Kim S.K., Kim N.H. (2006). Effects of the addition of multi-walled carbon nanotubes on the positive temperature coefficient characteristics of carbon-black-filled high-density polyethylene nanocomposites. Scr. Mater..

[35] Wang L., Hong J., Chen G. (2010). Comparison study of graphite nanosheets and carbon black as fillers for high density polyethylene. Polym. Eng. Sci..

[36] Dufresne A., Paillet M., Putaux J.L., Canet R., Carmona F., Delhaes P., Cui S. (2002). Processing and characterization of carbon nanotube/poly(styrene-co-butyl acrylate) nanocomposites. J. Mater. Sci..

[37] Balberg I., Azulay D., Toker D., Millo O. (2004). Precolation and Tunneling in composite Materials. Int. J. Mod. Phys. B.

[38] Kirkpatrick S. (1973). Percolation and Conduction. Rev. Mod. Phys..

[39] Trujillo M., Arnal M.L., Müller A.J., Bredeau S., Bonduel D., Dubois P., Hamley I.W., Castelletto V. (2008). Thermal fractionation and isothermal crystallization of polyethylene nanocomposites prepared by in situ polymerization. Macromolecules.

[40] Su Z., Li Q., Liu Y., Guo W., Wu C. (2010). The nucleation effect of modified carbon black on crystallization of poly(lactic acid). Polym. Eng. Sci..

[41] Szymczyk A.A., Roslaniec Z., Zenker M., García-Gutiérrez M.C., Hernández J.J., Rueda D.R., Nogales A., Ezquerra T.A. (2011). Preparation and characterization of nanocomposites based on COOH functionalized multi-walled carbon nanotubes and on poly(trimethylene terephthalate). Express Polym. Lett..

[42] Perez R.A., Lopez J.V., Hoskins J.N., Zhang B., Grayson S.M., Casas M.T., Puiggali J., Müller A.J. (2014). Nucleation and antinucleation effects of functionalized carbon nanotubes on cyclic and linear poly(ε-caprolactones). Macromolecules.

[43] Müller A.J., Arnal M.L., Trujillo M., Lorenzo A.T. (2011). Super-nucleation in nanocomposites and confinement effects on the crystallizable components within block copolymers, miktoarm star copolymers and nanocomposites. Eur. Polym. J..

[44] Zeng Y., Liu P., Du J., Zhao L., Ajayan P.M., Cheng H.-M. (2010). Increasing the electrical conductivity of carbon nanotube/polymer composites by using weak nanotube–polymer interactions. Carbon.

[45] Hong J., Park D.W., Shim S.E. (2012). Electrical, thermal, and rheological properties of carbon black and carbon nanotube dual filler-incorporated poly(dimethylsiloxane) nanocomposites. Macromol. Res..

[46] Wen X., Tian N., Gong J., Chen Q., Qi Y., Liu Z., Liu J., Jiang Z., Chen X., Tang T. (2013). Effect of nanosized carbon black on thermal stability and flame retardancy of polypropylene/carbon nanotubes nanocomposites. Polym. Adv. Technol..

[47] Lorenz H., Fritzsche J., Das A., Stöckelhuber K.W., Jurk R., Heinrich G., Klüppel M. (2009). Advanced elastomer nanocomposites based on CNT-hybrid filler systems. Compos. Sci. Technol..

[48] Zweifel H. (1998). Stabilization of Polymeric Materials.

[49] Gugumus F. (2001). Re-examination of the thermal oxidation reactions of polymers 1. New views of an old reaction. Polym. Degrad. Stab..

[50] Gugumus F. (2002). Re-examination of the thermal oxidation reactions of polymers 2. Thermal oxidation of polyethylene. Polym. Degrad. Stab..

[51] Bravo A., Hotchkiss J.H. (1993). Identification of volatile compound resulting from the thermal oxidation of polyethylene. J. Appl. Polym. Sci..

[52] Wen X., Wang Y., Gong J., Liu J., Tian N., Wang Y., Jiang Z., Qiu J., Tang T. (2012). Thermal and flammability properties of polypropylene/carbon black nanocomposites. Polym. Degrad. Stab..

[53] Galano A. (2008). Carbon nanotubes as free-radical scavengers. J. Phys. Chem. C.

[54] Azizi S., David E., Fréchette M.F., Nguyen-Tri P., Ouellet-Plamondon C.M. (2018). Electrical and thermal conductivity of ethylene vinyl acetate composite with graphene and carbon black filler. Polym. Test..

[55] Azizi S., David E., Fréchette M.F., Nguyen-Tri P., Ouellet-Plamondon C.M. (2019). Electrical and thermal phenomena in low-density polyethylene/carbon black composites near the percolation threshold. J. Appl. Polym. Sci..

[56] Song J., Li X., Ma L., Li W. (2019). YaoShichune Thermal conductivity of natural rubber nanocomposites with hybrid fillers. Chin. J. Chem. Eng..

